# The Medical Information Scientific Process: Define, Research, Evaluate, Synthesize, and Share (DRESS)

**DOI:** 10.1007/s43441-021-00366-w

**Published:** 2022-03-03

**Authors:** Dominick Albano, Farah Pragga, Rena Rai, Teresa Flowers, Prachi Parmar, Susan Wnorowski, Evelyn R. Hermes-DeSantis

**Affiliations:** 1 Pfizer Inc., 500 Arcola Road, Collegeville, PA 19426 USA; 2 Pharma Collaboration for Transparent Medical Information™, West Point, PA USA; 3 Pfizer Inc., 235 East 42 Street, New York, NY 10017 USA; 4 Rutgers, The State University of New Jersey, Piscataway, NJ USA; 5 Astellas Pharma Inc., 1 Astellas Way, Northbrook, IL 60062 USA; 6 Ipsen Biopharmaceuticals, Inc., Basking Ridge, NJ USA

**Keywords:** Medical information service, Medical information science skills, Medical information practice, Medical information inquiry handling, Literature review

## Abstract

Medical information (MI) professionals are primarily responsible for researching and responding to unsolicited requests for information on their company’s product(s). In an effort to set a standard for quality, the Pharma Collaboration for Transparent Medical Information (phactMI) created a code of practice for the provision of medical information to healthcare professionals. This code introduced the term “MI science skills” to describe the expertise required to perform the duties of an MI professional. These skills can be summarized by the acronym DRESS. In order to effectively and efficiently respond to an unsolicited request for information, the MI professional essentially follows five steps: define the question, research the topic, evaluate the evidence, synthesize a response, and share the answer. As this approach mirrors the scientific process for data generation, MI scientist may be a more apt description for this role. This paper explains the rationale behind the term MI scientist and the skills associated with each component of the DRESS approach.

## Introduction

Effectively responding to a medical information inquiry is akin to the scientific process [1]. The Pharma Collaboration for Transparent Medical Information™ (phactMI) was formed in December of 2014 to support the safe and effective use of medicines. One of the first acts of phactMI was to develop the “medical information code of practice (COP) for responding to healthcare professionals (HCPs) requests.” Published in 2015, the COP defined three fundamental elements for medical information (MI) practice: clinical and pharmaceutical expertise, scientific balance of medical responses, and quality standards. The COP also introduced the term MI science skills to describe the technical expertise required of MI practitioners. As stated in the code: “MI professionals should be trained in MI science skills, including literature searching, literature evaluation (i.e., study designs, statistical methodology, and clinical product/device training), information synthesis, and medical writing. MI professionals should be able to critically evaluate scientific literature, formulate a medical response, and effectively communicate the information to the requesting HCP to assist them in making a clinical decision [2].”

The term MI science Skills was selected because the process by which an MI professional fulfills their duties for responding to unsolicited medical information inquiries is generally analogous to the scientific process. Stated simply, the scientific method starts with a question, followed by experimentation, analysis/interpretation, and publication or communication of results [3]. Similarly, answering an MI inquiry starts with understanding the question, followed by obtaining relevant evidence to answer that question, then critically appraising or evaluating the evidence, and finally communicating an answer in writing or verbally. Each of the steps in the MI process requires certain expertise and hence the term MI science skills was born. By the same rationale, those practicing MI science skills can therefore be referred to as MI scientists. In this paper, we describe in more detail the aspects of these MI science skills exercised by MI scientists.

## Case Studies

The primary responsibility of an MI scientist is to research and respond to unsolicited requests for information on their company’s product(s). The inquiries MI scientists receive and respond to vary in terms of nature and complexity. Some questions are straightforward and tend to be answered directly from a product’s prescribing information. For example, Dr. Jones is a cardiologist requesting information regarding the recommended titration schedule for a patient starting product X, a hypertension management medication. Requests that are less complex in nature may also include topics, such as recommended dosing, mechanism of action, medication properties, and approved indications. Complex inquiries require more effort as the MI scientist must utilize their MI science Skills to research and develop a tailored response to the question at hand. Requests that are complex in nature can include topics, such as off-label use, alternative dosing and administration, use in special populations, use with other medications, and others. For example, Dr. Doe is a hospital pharmacist that works closely with his institution’s surgical team. He is requesting information regarding the use of product B, an intravenous corticosteroid, as an alternative for opioid analgesics in a 55-year-old male patient undergoing spinal surgery. As this information is not readily available, the MI scientist must utilize the skillset described in this paper by the acronym DRESS (define the question, research the topic, evaluate the evidence, synthesize a response, and share the answer) to research and respond to this inquiry.

### Define the Research Question

The role of an MI scientist requires responding to specific questions with truthful, non-biased, scientifically accurate, and current medical information. Their clinical expertise on the product(s) and specific therapeutic areas is required to help better understand and define the query. The MI role requires great attention to detail, so the response can be tailored to the specific question asked. Watanabe et al. presented a 5-step systematic approach for how to respond to drug information requests [4]. The five steps were to classify the request (for example: is this a safety question or a dosing question, or both?), obtain background information on the request, conduct a systematic search, develop a response, and reclassification (to determine if the response aligns with the initial classification of the request). Kirkwood modified the approach to 7 steps that involves the following: identify requestor demographics; obtain background information of request; determine and categorize the specific question; develop and implement a search strategy; evaluate, analyze, and synthesize the data; formulate and provide a response; and follow-up and document [5]. Using a systematic method supports answering a complex and difficult question with an appropriate response. Straightforward questions also need further clarification, as there may be more background information to help better answer the ultimate question.

It is essential to know the requestor’s demographics. This may provide insight into the requestor's level of sophistication and knowledge regarding the subject matter. For example, is it a patient or a pharmacist or a physician? Understanding the context of the question can help to better formulate an appropriate response [4, 5]. The MI scientist asks the appropriate clarifying questions that can lead to a deeper understanding of the true nature of the requestor’s inquiry and confirms the research question. The MI scientist realizes that the query may not have been asked very concisely or clearly. They also recognize that the responses to the query may differ depending on the patient’s medical history or other special circumstances. There are two key aspects to understanding the question: (1) It is important to find out if the question is specific to a patient’s condition or if it is truly a question for the requestor’s own knowledge. (2) It is also good to know what resources or what search strategies the requestor already used. This can avoid duplication of effort or an opportunity to clarify misinformation and validate appropriate sources of information [4–6].

Suppose the requestor asks a general question on the dosing for a drug. In this case, it is important for the MI scientist to further clarify the context, especially for a drug that may have multiple indications. It is best practice to confirm with the requestor the ultimate question before the MI scientist creates the search strategy [4, 5].

### Research the Topic

The ability to conduct an effective literature search is one of the critical steps in achieving competency in evidence-based medicine and a key competency for an MI scientist [7]. Once a well-defined research question has been created, the next step is to create an effective search strategy by which to find the data to answer the question. The strength of a scientific response depends on the data used to support it. Literature stressing the importance of searching skills can be found in publications on evidence-based practice in medicine [7–9]. Although there are a limited number of studies, there is evidence that many healthcare professionals feel they could be more proficient in literature searching and that formal training may improve overall search and retrieval skills [9, 10].

A search strategy encompasses several steps and considers the requestor, the breadth of the topic, and the availability of databases. Based on the research question, the MI scientist will identify the search terms to use and how these terms will be combined. For example, will the search use a Boolean approach (OR, AND, or NOT)? Alternatively, will free text be used or will a closed vocabulary such as medical subject heading (MeSH) terms be applied [11, 12]? Various searching tools can assist in the development of appropriate search strategies. For example, the PICO tool emphasizes searching by the population, intervention, comparison, and/or outcomes, while the modified PICOS tool adds in study design [13]. Based on the MI scientist’s skills and knowledge, the search strategy will be developed and executed. Most commonly used databases provide a search-builder function to assist the researcher [11, 12].

There are numerous databases of medical literature, each designed with a specific purpose. See Table 1 for examples of some commonly used literature retrieval databases [14–19]. An MI scientist may also refer to guidances set forth by regulatory bodies, such as the US Food and Drug Administration (FDA) and the European Medicines Agency (EMA) as reputable sources of information. By becoming familiar with available sources, the MI scientist can develop a more effective search strategy. In the current era, we are fortunate to have access to many free and subscription-based high-quality electronic databases. A particular institution or company may subscribe to one or more of these databases and it is important to be acquainted with each one to understand what they offer [20]. It is also important to only utilize reputable databases to avoid misinformation such as evidence that may exist in predatory journal articles which have not undergone the same peer-review scrutiny [21].

**Table 1:** Examples of commonly used literature retrieval databases [14–19]

Database name	Content	Details	Limitations (11)	Vocabulary
Public databases				
Medline [19]	More than 26 million references in life sciences with a focus on biomedicine	US National Library of Medicine database. Historically focused on US content, although expanding. Primary component of PubMed	Subscription required; difficult to retrieve references not yet indexed	Indexed with NLM Medical Subject Headings (MeSH®)
PubMed [17]	Free resource with more than 30 million references. Does not contain full text but might link to full text	Considered a partner with Medline. Recently updated to be mobile friendly	Does not contain full text but might link to full text. Excludes most of the literature that is not peer reviewed (gray literature) which may result in an incomplete search	MeSH or free text (open vocabulary)
Google Scholar [18]	Literature in any language uploaded to the Internet in electronic format	From parent company Google	Compared to other databases, results can be skewed by number of visitors rather than relevance, less frequently updated	Natural language
Subscription-based databases				
EMBASE [14]	More than 37 million records, including more than 6 million records and close to 3000 journals not covered by Medline	Owned by Elsevier, greater focus ex-US so could be considered companion to Medline/Pubmed for global coverage. Extensive coverage of congresses and medical device literature	Only available by subscription	Indexed with embase indexing and emtree
OVID [16]	More than 6,000 eBooks, over 1400 premium, peer-reviewed journals-with no embargoes (with one exception—science). Over 100 bibliographic and full-text databases	Owned by Wolters Kluwer. Provides database offerings in Chinese, French, and Spanish. Offers a dedicated work area into which a researcher can organize and manage material	Boolean searches default to keyword searches which may result in less robust retrieval results	Natural language searching
Web of science [15]	Web of science is a platform consisting of several literature search databases (including Medline) Includes > 21,419 journals + books and conference proceedings. Over 79 million records More than 119,000 books Over 220,000 conferences covered	Held by clarivate analytics. Has language and geography-focused options, for example, Chinese Science Citation database, Russian Science Citation Index, and Korean Journal Index. The core collection serves as the standard dataset underpinning journal impact metrics found in the journal citation report	Only available by subscription	No controlled vocabulary. Keyword fields include author keywords and “Keywords +” which are extracted from the titles of cited articles. Controlled indexing is provided for institution affiliations

MI scientists may take advanced biomedical literature search training to enhance their skillset; this may include connecting with a skilled librarian who can provide instruction on working with various databases, using indexing, and developing advanced search strategies. For non-governmental databases to which an organization subscribes, it is often possible to contact the owner of the database for training. Once the MI scientist has conducted a thorough literature search, the next step in the process is to evaluate the literature.

### Evaluate the Evidence

Critical appraisal/literature evaluation skills are key competencies for an MI scientist. Depending on the nature of the medical inquiry, there may be a wide variety of literature available. It is important to remember that not all study designs are created equal. Hierarchies of literature can be found in a variety of references and resources. The Oxford Centre for Evidence-Based Medicine provides a thorough background and explanation of their version of the levels of evidence [22–24]. Based on the question that is being answered, levels of evidence can differ. It is essential to utilize the highest level of data available to answer a question. For example, if there are plenty of available randomized controlled trials, it is unnecessary to summarize lower quality trials or case reports/series. However, if there is only observational data available, then the response document would be reflective of that [25]. The MI scientist must be familiar with the levels of evidence and types of study designs. Table 2 provides a high-level summary for easy reference.

**Table 2:** Study designs, levels of hierarchy, and important considerations [22–24]; [35–37])

Level	Types of Study Designs/Trials	Description	Considerations
1	Systematic reviews of randomized controlled trials	An integration of statistically evaluated results that come from previously conducted studies. This type of design considers an individual study as the unit of analysis vs the individual patient.	•Systematic reviews should be used over individual trials when available; highest standard of literature •Could be useful when past studies may have had inconsistent results and included smaller number of subjects
2	Randomized trial or observational study with dramatic effect	A trial with both an experimental (interventional) and a control (placebo or standard treatment) group in which patients are randomly assigned; can be parallel or cross-over Real world evidence or comparative effectiveness trials may be included	•Randomized trials are considered the gold standard, strongest study design, and highly reliable for majority of medical questions •Sample should be representative of the population that the intervention would apply to •Real World Evidence studies often seek effectiveness data in clinical settings, accounting for variables, such as adherence, switching, discontinuing, and/or concomitant medications. These studies are considered complementary to randomized controlled trials as they often fill data gaps left by randomized trials
3	Non-randomized controlled cohort/follow-up study	Strongest observational study where subjects that are free of the outcome are divided into groups: one that is exposed to the factor of interest and the other that is not exposed. Groups are not randomly assigned in this design. Both groups need to have standardized eligibility criteria and outcome assessments	•Reliable and applicable for questions related to diagnosis, prognosis, and incidence. May not be best for questions of efficacy •A limitation is that these studies tend to be lengthy and a large number of subjects are involved •There could be a lack of randomization, difficulty in identifying controls, and hidden confounding factors linked to the exposure
4	Case-series, case–control studies, or historically controlled studies	Case control: helps to determine if a particular exposure is associated with an outcome. Patients with a specific outcome of interest (disease or condition) are matched with patients that do not have that same outcome of interest. Investigators look back in time to determine frequency of exposure between the two groups Cross-section: examines the relationship between health-related characteristics or diseases and other variables of interest that exist in a defined population at a single point in time Case report: descriptive report of an observation made on a specific patient or on multiple patients (i.e., adverse event). Retrospective in nature	•Less reliable due to potential for recall bias and retrospective data collection (i.e., inconsistency in patient records) •This design might be beneficial when there is a long-lag time between exposure and outcome
			•Study design helps to establish association but not causality. Disadvantages are recall bias and inequitably distributed confounders
			•May be challenging to interpret outcomes due to multiple factors; however, could be useful if other types of studies are unavailable
5	Mechanism based reasoning		•Regardless of the medical question being addressed, this is the least reliable information and based mainly on expert opinion without explicit critical appraisal

Based on the quality of any of the studies, the level of evidence can be shifted down. It can also be shifted up if there is a large/very large effect size

Additionally, most MI departments have their own guidance documents and standard operating procedures (SOPs) around the development of a written scientific response. These guidance documents provide a framework of elements to include in each section of a scientific response document. Based on the inquiry, not all elements are necessary for all documents. Realistically, it is not possible or always appropriate to include all publications from a literature search. In fact, an MI scientist needs to use their professional expertise and judgment to develop concise, accurate, scientifically balanced, non-promotional, and evidence-based scientific response documents.

Once all relevant references are retrieved, a critical evaluation is next. However, most busy practicing HCPs, including those in the pharmaceutical industry, do not have the luxury of time to critique each article in a “journal club” fashion. While it is important for MI scientists to have a working knowledge of appropriate reporting items for various trial designs, practically speaking, it is helpful to have a concise and quick method to critically evaluate the literature. Table 3 summarizes the brief questions that help in evaluating a clinical trial [26–28].

**Table 3:** Questions to ask when summarizing a clinical trial [26–28]

Section	Questions	Comments
Introduction	What is the objective of the study?	This sets the stage for everything else in the study
Methods	What is the study design?	
	Are inclusion criteria adequate?	The predefined criteria for patients to participate in the clinical trial should be clearly specified and appropriate so that the study is clinically relevant to the question being asked
	Are exclusion criteria adequate?	The primary goal of exclusion criteria is to ensure patient safety. Investigators can use this criterion to exclude patients that might have a higher risk of increased adverse events or other safety issues. On the other end, exclusion criteria shouldn’t be so restrictive that the study results are not clinically relevant to a specific patient population that would require treatment
	Is the study randomized?	Randomized trials are considered the gold standard and highly reliable for majority of medical questions
	Is the length of the study appropriate to show effect?	If a study hasn’t been conducted for an appropriate amount of time, then a treatment effect may not be seen when in fact there is potential for this effect
	Are dosages and treatment regimens appropriate?	The dosage and treatment regimen being used in the study should be representative of what is typically observed for the specific indication being addressed
	Is the study blinded?	Compared to a single-blinded study, a double-blinded study makes an effort to prevent investigator and subject bias. If there are barriers to blinding, then that could be a major limitation of the study
	Are the endpoints standard, validated, or accepted?	The test or measurement of evaluation used in the study to accurately assess the primary outcome needs to be either formally validated or an accepted practice. Otherwise, even statistically significant results may not be relevant and could leave a doubt in the reader's mind
Statistics	Are the statistics appropriate?	
	Is power set and/or met?	Power, or the risk of avoiding a type II error, is critical for evaluating a study. The lowest acceptable power is 80%, if the power is not set or met, it may mean that the study does not have enough enrolled subjects to detect a difference
	Delta	The clinically relevant difference being sought. There should be an explanation as to why the number was selected
	Are appropriate statistical tests used for type(s) of data	The statistical test is selected based on the type of data collected for the endpoint of the study
Results	Demographic	
	Did randomization result in similar groups?	After the subjects are randomized, each study group should have similar baseline characteristics and demographics. If there is significant inequality between treatment groups, it is unclear if the results observed were due to this difference in groups or the actual study drug
	How similar is the study group to typical patient group with the disease?	The group being studied should appropriately reflect the demographic characteristics (i.e., age, gender, and race) of the patient population with the disease state
	Efficacy results	The meat of the study. This section needs to be evaluated for both statistical significance and clinical relevance. All endpoints mentioned in the methods should be accounted for
	Safety results	As a balance to the efficacy, safety data need to be evaluated as well
Conclusions		
	Are they supported by the results?	The results and data should match what the author is stating as the conclusions of the study. If there is a discrepancy between the results and the conclusion, it could lead the study to be potentially questionable

The statistical analysis section of the article is usually overlooked due to a poor understanding of statistics [29]. While most individuals remember that the *p* value needs to be below 0.05 to be considered statistically significant, few realize this is an arbitrary number and there is a push to move away from a *p* value of 0.05 [30, 31]. In evaluating a clinical trial results, it is important to not only look at statistical significance but also the clinical relevance of the results. Is there a ‘so what?’ For example, based on the question posed in the case studies section of this article, if intravenous corticosteroids could statistically significantly reduce the use of opioids post-surgery, you would likely recommend them. However, if the reduction was only 0.5 mg morphine over a 72-h period, while this may be statistically significant, it may not be clinically relevant. So, the response should mention the decrease, but highlight the limitations.

### Synthesize the Response and Share the Answer

Professional medical writing and verbal communication skills are important for the delivery of a MI response to help the HCP make an informed clinical decision. This includes restating the relevant background information and providing a summary of the literature, search strategy, and references used [32].

Recently, phactMI published a proposed best practice guideline that details the sections of a scientific response document [25]. The MI scientist can use these best practices to present the written response documents in a consistent format that is user-friendly for the intended audience. The methods and types of communications are tailored to the individual based on factors such health literacy and depth of knowledge about the question at hand. Each response is curated to meet the unique needs of the requestor, whether he or she is a patient, caregiver, or HCP and with consideration for their level of specialization and knowledge. An MI scientist understands that they do not provide recommendations or advice to HCPs or patients. Their responses should be written in a way that does not persuade the reader to follow a specific course of action. The responses should be factual, objective, and only consist of scientifically balanced information identified in the literature search or other appropriate internal sources (data on file) [32].

When an MI scientist uses their therapeutic area expertise and knowledge on drug therapy to respond by phone or in-person (e.g., at a Medical Congress), it is essential to demonstrate professional courtesy, empathy, and employ active listening techniques. As the MI scientist prepares a narrowly tailored-specific response, they also must be prepared for additional follow-up queries.

The MI scientist is a customer-facing role where they are acting on behalf of the company. The MI scientist has a professional and ethical responsibility to provide evidence-based, scientifically balanced, and non-misleading answers to MI requests [32, 33].

## Discussion

Medical information professionals in the pharmaceutical industry have a unique responsibility to provide accurate, unbiased, evidence-based, and scientifically balanced information about their company’s product(s) to patients and HCPs. The phactMI code of practice (COP) describes MI science skills as the technical acumen that medical information professionals are equipped with to carry out their responsibilities effectively [2]. Aside from their technical skills, MI professionals often hold advanced healthcare degrees and have relevant clinical expertise [34]. Given the scientific and methodical nature of their work, MI scientist may be a better descriptor for the role.

This skillset that MI scientists must utilize can be described by the acronym DRESS (defining the question, researching the topic, evaluating the evidence, synthesizing a response, and sharing the answer). The rigorous process by which MI scientists respond to unsolicited medical information requests parallels the scientific method. Every scientific experiment starts with a question or hypothesis, for example, is drug A safe and effective in treating condition B? To ensure full comprehension of the topic, the scientist must have background knowledge through research of the subject. The necessary background can help define the confounding variables. Next, the researcher must design an experiment and collect the required data. They then carefully evaluate the data and determine the answer to the research question based on the evidence. After drawing a conclusion, the researcher may choose to communicate the findings in a scientific report.

MI scientists work in a similar manner. When Dr. Doe, a hospital pharmacist, sends a request to the medical information department responsible for product B, five key steps are undertaken to send him an accurate and scientifically balanced response. These steps are similar to the scientific method:

1. The MI scientist begins the process by *defining the question*. Often this step requires background knowledge of the topic.
2. Once the question is well defined, the MI scientist *researches the topic* in the biomedical literature for relevant data. MI scientists are well versed in utilizing different databases to build a robust search strategy for a given topic.
3. After gathering the available data, the next step is to *evaluate the evidence*. MI scientists use their expertise to evaluate and critically appraise clinical data, keeping in mind that not all levels of evidence are the same. Utilizing their knowledge of the literature and professional judgment, MI scientists respond to inquiries with the highest level of evidence available.
4. Once the evidence has been evaluated, the next step is *synthesizing a response*. This requires the skill and understanding of who is asking the question and the channel through which the response will be distributed.
5. The final step then is *sharing the answer* with the requestor in a clear, accurate, balanced, and succinct manner.

Using the DRESS approach can organize the process by which MI scientists make available the right information to clinicians in a format for decision-making.

## Conclusion

Professionals in the medical information departments of pharmaceutical companies are highly trained individuals uniquely positioned to provide accurate and scientifically balanced information about their drug(s). The methodological approach followed by MI scientists to respond to MI requests parallels the scientific method. Following the DRESS approach, the MI scientist can effectively and efficiently respond to the information needs of customers, thereby supporting optimal patient care and outcomes.
